# Supplementary material for: Predicting mean depth and area fraction of Antarctic supraglacial melt lakes with physics-based parameterizations
Source: Nat Commun. 2025 Jul 15;16:6518. doi: 10.1038/s41467-025-61798-8 (PMC12263893; doi:10.1038/s41467-025-61798-8)
Supplement: Supplementary file 1 — Supplementary Information [file 41467_2025_61798_MOESM1_ESM.pdf]

Predicting Mean Depth and Area Fraction of Antarctic Supraglacial Melt Lakes with Physics-Based Parameterizations Supplementary

Danielle Grau, Azeez Hussain, Alexander A. Robel *

June 25, 2025

Spatial Roughness of ICESat-2 Intersections

ICESat-2 tracks cover a vast majority of the Antarctic continent, measuring the ice sheet's surface height. The satellite's path is known to cross over sections of topography at different angles, which has raised the question of how the calculated surface roughness values of the topography change with the satellite's orientation. We use the existing analyzed ICESat-2 sub-tracks to identify those that intersect each other (have angle difference greater than 90°) and compare the roughness properties between them.

It is evident from Figure 1 that the Hurst exponent doesn't change with the capture angle of the satellite. Nearly all of the calculated values are center of the continental average of Antarctica of 0.4. It is independent of the orientation or viewpoint of the surface and is an intrinsic characteristic of the surface itself. The same cannot be said for the standard deviation of topography. The standard deviation very much changes with the orientation of the satellite as it captures different sections of topography across the surface.

*School of Earth and Atmospheric Sciences, Atlanta, GA

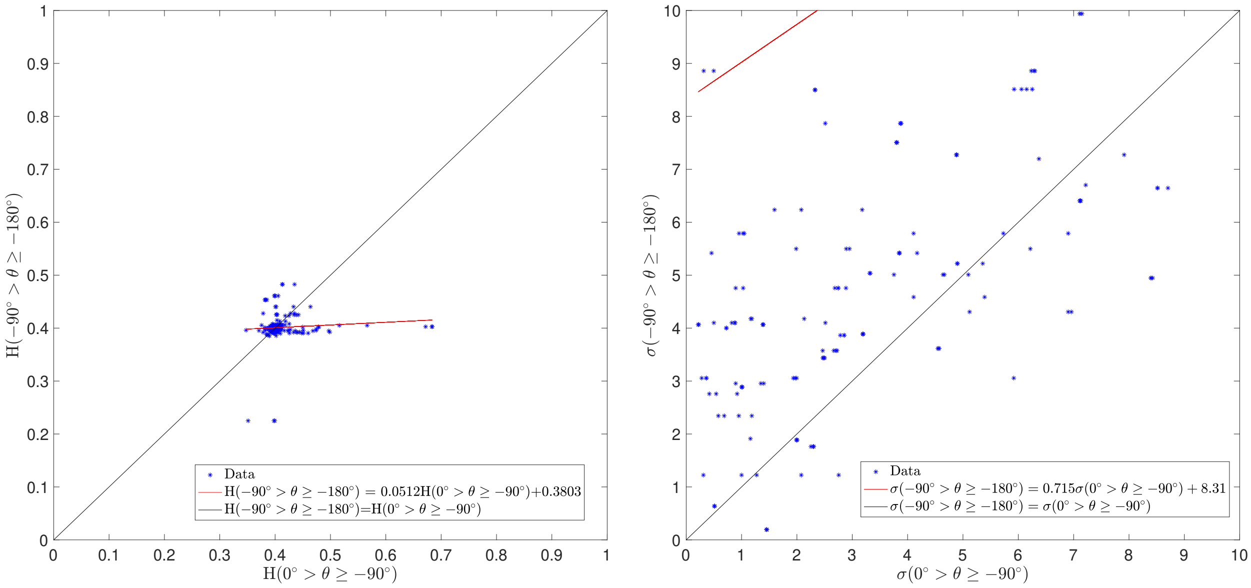

Supplementary Figure 1: **Difference in calculated Hurst exponent and standard deviation of topography between different Icesat-2 track angles. (Left)** The blue points are the calculated hurst exponents of intersecting ICESat-2 sub-tracks with normal angle to each other. The black solid line is the one-to-one relationship between hurst exponent values, and the red line is the fitted linear regression of the data. **(Right)** Similarly to the left panel, the blue points are the calculated standard deviation of topography of intersecting ICESat-2 sub-tracks with an angle difference greater than 90°. The solid black line is the one-to-one relationship between standard deviation values and the red line is the fitted linear regression.

Mean Water Depth Parameterization

In addition the two melt lake characteristics (average lake depth ($\bar{w}_l$) and average fraction area ($\bar{F}$), we have an additional parameter known as the average water depth ($\bar{w}_d$) which the average depth of water across a surface. The average water depth has a direct relationship with the aforementioned melt lake parameters (Equation 6).

As described in the main text, the same process for developing the other parameterizations is done for the mean water depth (($\bar{w}_d$). The resulting parameterization fits the numerical data with a R^2 of 0.78. This fit is the poorest among the three parameterizations, but as expected the main trends observed in the numerical results are captured in the parameterizations.

$$\bar{w}_d = 0.9\sigma \operatorname{erf}(0.27S)(1 - 0.08H^{0.6} - 0.8 \operatorname{erf}(0.76S)) \quad (1)$$

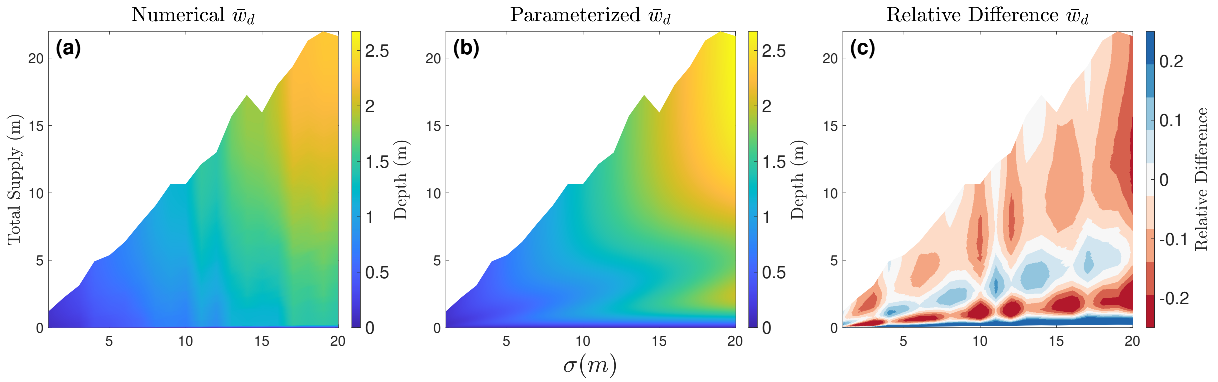

Supplementary Figure 2: **Mean supraglacial melt water depth simulation results.** **a.** Parameter space of the mean water depth-averaged over 500 randomly generated self-affine surfaces. **b.** The parameter space of the mean water depth of parameterizations fits with the input variables of the numerical simulations. **c.** The relative difference between the numerical and predicted mean water depth. The Hurst value is held constant at 0.4 for this parameter space.

26 # Additional Figures

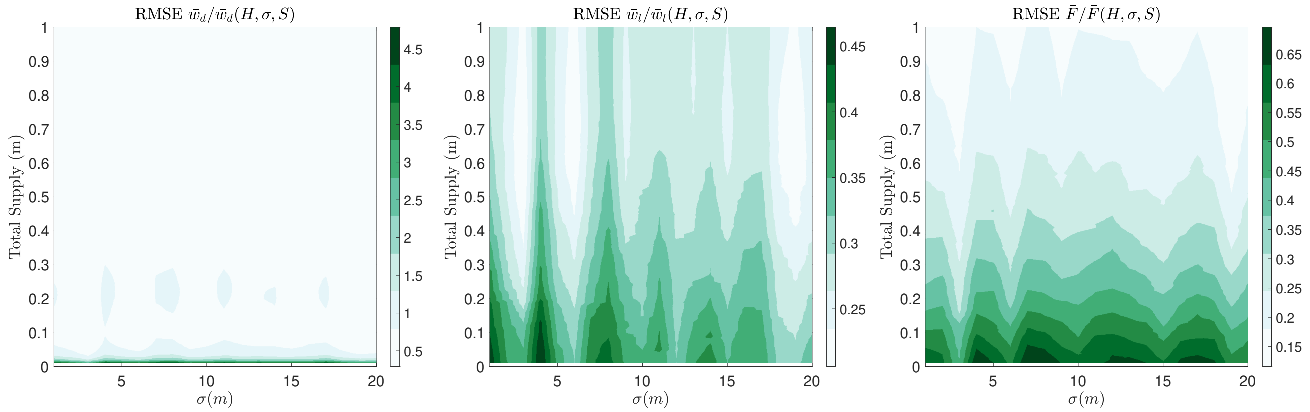

Supplementary Figure 3: **Root-Mean Square Error of the supraglacial melt lake parameterizations.** The Root-Mean Square Error divided by the parameterization for the three melt lake characteristics The root mean square error is between the estimated value of the melt lake characteristics from the parameterizations and every instance (every generated surface) from the numerical simulations.

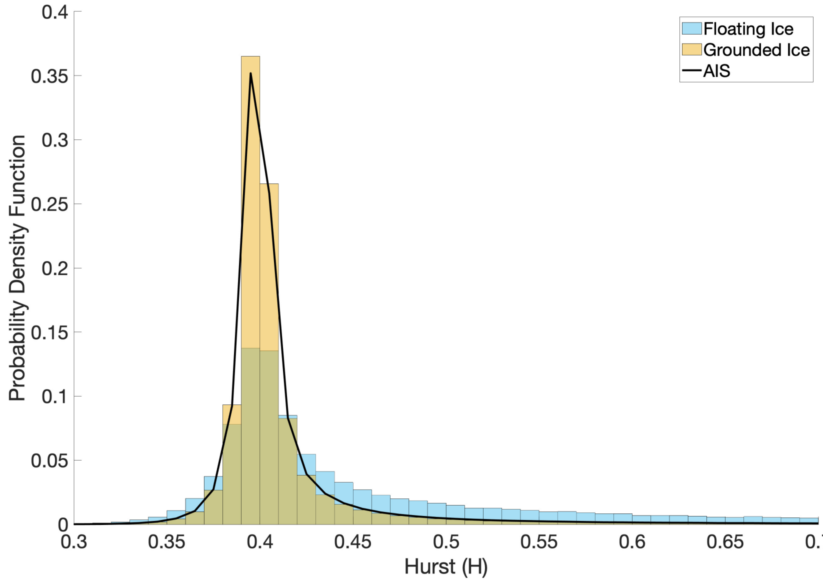

Supplementary Figure 4: **Distribution of Hurst Exponents (H) across the Antarctic Ice Sheet.** The Probability Density Function of the hurst exponent of floating ice (blue) and grounded ice (yellow). The solid black line is the probability density of the entire continent. The hurst exponent of floating ice has a wider distribution in comparison to the grounded ice.

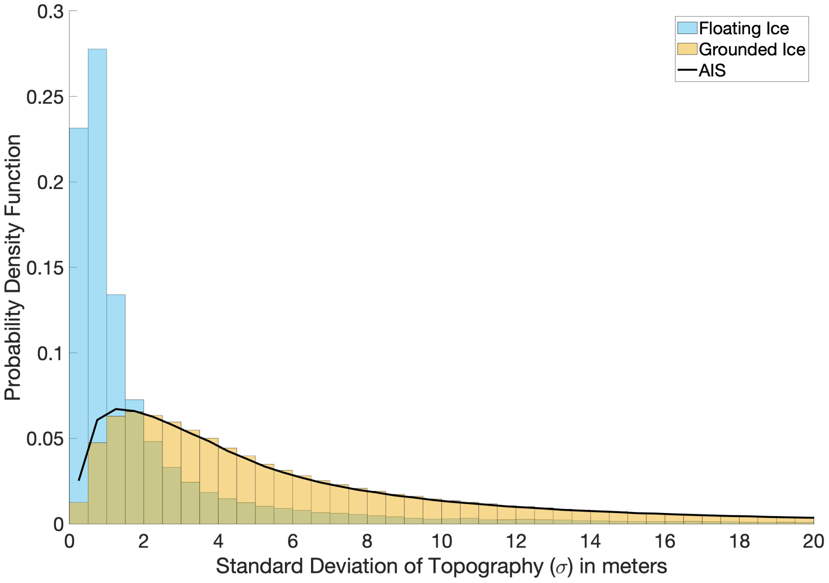

Supplementary Figure 5: **Distribution of the Standard Deviation of Topography (σ) on the Antarctic Ice Sheet.** The Probability Density Function of the standard deviation of topography of floating ice (blue) and grounded ice (yellow). The solid black line is the probability density of the entire continent. The standard deviation of topography of floating ice has a higher propensity to be less than 2 meters in comparison to grounded ice.
